# Conformation and Stability of Intramolecular Telomeric G-Quadruplexes: Sequence Effects in the Loops

**DOI:** 10.1371/journal.pone.0084113

**Published:** 2013-12-18

**Authors:** Giovanna Sattin, Anna Artese, Matteo Nadai, Giosuè Costa, Lucia Parrotta, Stefano Alcaro, Manlio Palumbo, Sara N. Richter

**Affiliations:** 1 Department of Molecular Medicine, University of Padua, Padua, Italy; 2 Dipartimento di Scienze della Salute, Università "Magna Græcia" di Catanzaro, Catanzaro, Italy; 3 Department of Pharmaceutical and Pharmacological Sciences, University of Padua, Padua, Italy; INSERM UMR S_910, France

## Abstract

Telomeres are guanine-rich sequences that protect the ends of chromosomes. These regions can fold into G-quadruplex structures and their stabilization by G-quadruplex ligands has been employed as an anticancer strategy. Genetic analysis in human telomeres revealed extensive allelic variation restricted to loop bases, indicating that the variant telomeric sequences maintain the ability to fold into G-quadruplex. To assess the effect of mutations in loop bases on G-quadruplex folding and stability, we performed a comprehensive analysis of mutant telomeric sequences by spectroscopic techniques, molecular dynamics simulations and gel electrophoresis. We found that when the first position in the loop was mutated from T to C or A the resulting structure adopted a less stable antiparallel topology; when the second position was mutated to C or A, lower thermal stability and no evident conformational change were observed; in contrast, substitution of the third position from A to C induced a more stable and original hybrid conformation, while mutation to T did not significantly affect G-quadruplex topology and stability. Our results indicate that allelic variations generate G-quadruplex telomeric structures with variable conformation and stability. This aspect needs to be taken into account when designing new potential anticancer molecules.

## Introduction

**Figure 1:**
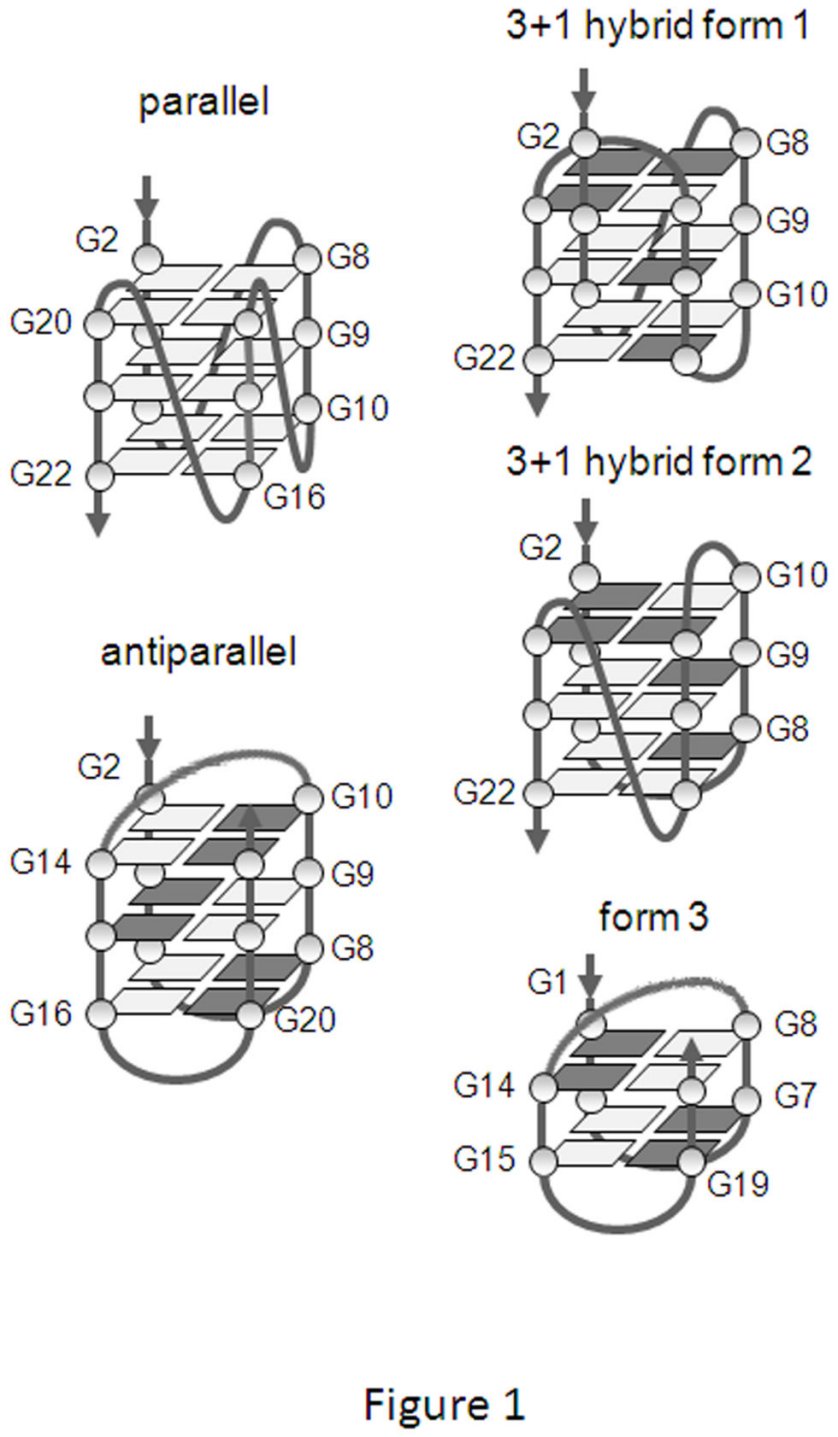
Model structures of conformations reported for the human telomeric sequence. Guanosines in dark grey are *syn*, and those in light gray are *anti*.

Telomeres are tandem repeats of guanine (G)-rich sequences that form the end of linear eukaryotic chromosomes, protecting them from degradation and repair activities, and therefore ensuring chromosome stability [1]. In humans, and in general in vertebrates, the repetitive canonical DNA telomeric sequence is 5’-TTAGGG-3’. However, detailed genetic analysis revealed extensive allelic variation at the proximal end of human telomeres, indicating a high underlying mutation rate [2,3]. Variant sequence repeats, such as CTAGGG [4], TCAGGG, TGAGGG [2] TTGGGG, and TTTAGGG [5] have been reported. In addition, repeats with unknown sequences and rarely with a change in the 6-bp periodicity have been observed. In all cases, the variants have shown a strong tendency to cluster, thus producing runs of the same sequence [2]. One common feature observed in the reported variants is that mutations were always restricted to non-G bases, therefore preserving the ability of the variant sequences to adopt a G-quadruplex conformation. G-quadruplexes are three-dimensional four-stranded structures of nucleic acids that can form in G-rich nucleic acids sequences. They consist of a square arrangement of Gs (G-tetrad), stabilized by hydrogen bonding. They are further stabilized by the presence of monovalent cations (preferably K^+^) in the center of the tetrads. G-quadruplexes can vary in a number of ways, including strand stoichiometry and strand orientation. In telomeres, intramolecular G-quadruplexes can form: depending on the direction of the parts of the strand that form the tetrads, structures are classified in three groups: parallel (group I), mixed or hybrid (group II) and antiparallel (group III) [6]. The wild-type (wt) human telomeric sequence has been shown to adopt all the three described topologies, depending on the conditions used for the analysis: antiparallel in Na^+^ solution [7], mixed “3+1 hybrid” in K^+^ solution [8], and parallel in crystals in the presence of K^+^ [9] (Figure 1). In the intramolecular G-quadruplexes, there are loops connecting different runs of guanines: these loops play a very important role in controlling the details of the resulting G-quadruplex structure and stability [10]. In the case of the four-repeat human telomeric DNA sequences, the same mixed “3+1 hybrid” structure has been reported to involve different loop arrangements in solution (hybrid form 1, hybrid form 2 and form 3) [11-13] (Figure 1).

Understanding the folding complexity at the telomere level has important therapeutic implications because stabilization of the telomeric G-quadruplex by reversible ligands has been shown to inhibit the activity of telomerase [14-16]. Several hundreds of small molecules that interact with the telomere have now been described in the literature. Their binding *in vitro* to human telomeric G-quadruplexes has been extensively explored, although cellular and *in vivo* data are only available for a small number of compounds [15]; in particular, the cyclic polyoxazole natural product telomestatin [17], and the synthetic acridine compounds BRACO19 [18] and RHPS4 [19] have shown encouraging results against xenograft cancer models and the fluoroquinolone derivative quarfloxin progressed to Phase II clinical trials for cancer [20]. In addition, the conformational knowledge about DNA G-quadruplex as a target allows performing structure-based rational discovery and optimization of scaffolds able to bind it, as reported by us in the case of psoralen [21] and fluorenone [22] derivatives.

Naturally occurring mutations at the telomeric repeats have been shown to be confined to the loop region [2]: therefore, while the ability to fold into G-quadruplex is conserved, different G-quadruplex topologies may be envisaged. Indeed, in the case of the CTAGGG sequence variant, NMR analysis in K^+^ solution proved the folding into an unexpected antiparallel G-quadruplex, with two G•G•G•G tetrads and one G•C•G•C tetrad [23]. Other reports investigated the conformational organization and stability of newly designed intramolecular G-quadruplexes, not necessarily derived from the human telomeric sequence, which maintained the four G-tracts while modified loop size and composition [24-27], or investigated modification of the G portion [28-31].

Here we performed a systematic analysis of loop nucleotide substitutions in the telomeric sequence, which supplement previous reports. The conformation and stability of the mutant telomeric sequences were tested by CD and UV spectroscopy, molecular dynamics simulations and protection assays. We found that both the nature of the base and the position within the loop control the resulting G-quadruplex conformation. In particular, modifications in the first and third positions in the loop remarkably affect G-quadruplex stability. 

## Materials and Methods

### Circular dichroism analysis

All DNA oligonucleotides were diluted from stock to final concentration (4 μM) in lithium cacodylate buffer (10 mM, pH 7.4) and KCl (50 mM). All samples were annealed by heating at 95 °C for 5 min, gradually cooled to room temperature and measured after 24 h. Compounds at 16 μM final concentration were added after DNA annealing. CD spectra were recorded on a Jasco-810 spectropolarimeter (Jasco, Easton, MD, USA) equipped with a Peltier temperature controller using a quartz cell of 5-mm optical path length and an instrument scanning speed of 100 nm/min with a response time of 4s over a wavelength range of 230-320 nm. The reported spectrum of each sample represents the average of 2 scans at 20°C and is baseline-corrected for signal contributions due to the buffer. Observed ellipticities were converted to mean residue ellipticity (θ) = deg × cm^2^ × dmol^−1^ (mol. ellip.). For the determination of T_m_, spectra were recorded over a temperature range of 20-95 °C, with temperature increase of 1 °C/min. T_m_ values were calculated according to the van’t Hoff equation, applied for a two state transition from a folded to unfolded state, assuming that the heat capacity of the folded and unfolded states are equal [32].

### Thermal difference spectrum (TDS) analysis

All DNA oligonucleotides were diluted from stock to final concentration (4 μM) in lithium cacodylate buffer (10 mM, pH 7.4) and KCl 50 mM. All samples were annealed by heating at 95°C for 5 min, gradually cooled to room temperature and measured after 24 h. UV spectra were recorded on Lamba25 UV/Vis spectrometer (Perkin Elmer) equipped with a Peltier temperature controller using a quartz cell of 10-mm optical path length and an instrument scanning speed of 600 nm/min over a wavelength range of 220-320 nm. UV spectra were recorded over a temperature range of 20-95°C. A 5 min equilibration period at each measurement was allowed to ensure homogeneous sample temperature. The autozero function was applied on the corresponding buffer at 20°C. TDS spectra were calculated by subtracting the spectrum below the melting (i.e. 15°C) from the spectrum above the melting (i.e. 95°C). TDS factors were calculated as the absolute values of Δ*A*_240nm_/Δ*A*_295nm_, where Δ*A*_*λ*_ is the difference, at the given wavelength λ, between the absorbance above (95°C) and below (15°C) the melting.

### Clerocidin protection assay

All oligonucleotides were gel-purified before use and prepared in desalted/lyophilized form. Oligonucleotides were 5’-end labelled with [γ-^32^P]ATP by T4 polynucleotide kinase and purified by MicroSpin G-25 columns. They were resuspended in annealing buffer (lithium cacodylate 10 mM, pH 7.4, with or without KCl 50 mM), heat-denatured and folded. 

Clerocidin reactions with the labelled oligonucleotides (4 pmol/ sample) were performed at 37°C in annealing buffer for 24 h. Samples were precipitated with ethanol to eliminate non-reacted drug, resuspended and either kept on ice, or treated at 90°C for 30 min with 1M piperidine. Samples were lyophilized, resuspended in formamide gel loading buffer, and heated at 95°C for 3 min. Reaction products were analyzed on 20% denaturing polyacrylamide gels and visualized by phosphorimaging analysis.

### Molecular modeling analysis

The PDB X-ray structure 1KF1 [9] and the NMR models 143D [7], 2HY9 [33], 2JPZ [34], 2JSL and 2JSM [12] related to the telomeric sequence *d*[AG_3_(T_2_AG_3_)_3_], were downloaded from the Protein Data Bank [35]; http://www.rcsb.org/pdb} to analyze the G-quadruplex structures in wt sequence. The two K^+^ ions, arranged in a square antiprismatic coordination, were placed between the stacked G-quartets and the same procedure was carried out with Na^+^.

In order to select the mixed model to use as starting point for our calculations, we evaluated the energetic stability of globally 80 receptor structures, since we included both original and optimized experimentally determined conformations of 2HY9 (10 original structures + 10 energy optimized structures), 2JPZ (10 original structures + 10 energy optimized structures), 2JSL (10 original structures + 10 energy optimized structures) and 2JSM (10 original structures + 10 energy optimized structures) NMR models. The hybrid structures 2HY9 and 2JPZ resulted both formed by 26-mer, while in the hybrid models 2JSL and 2JSM were reported sequences with, respectively, 25- and 23-mer. Thus, to obtain a similar analysis with respect to the first two models, the hybrid PDB structures were modified by deleting these caps, that is, considering them as conformational templates for the canonical 22-mer *d*[AG_3_(T_2_AG_3_)_3_]. All the structures were submitted to 3000 iterations of full minimization, using the Polake-Ribiere Conjugated Gradient (PRCG) algorithm, AMBER* [36] as force field with the “all atoms” notation, the implicit model of solvation GB/SA water [37] and the formal charges for all receptors as implemented in MacroModel ver. 7.2 [38,39] By analyzing all the obtained energy values, reported in Tables S1-S4 in File S1, we selected as wt G-quadruplex mixed form the most stable hybrid 2HY9 model. With the aim to better explain the position of the coordinating cations during the simulations, for each guanine plane of wt models we monitored 8 dummy angles centering K^+^ or Na^+^ with respect to N7 of non-adjacent guanines per quartet, as reported in Figures S1-S6 in File S1. Moreover, in order to display the antiprismatic coordination, we included an additional figure, generated from the last snapshot of the molecular dynamics simulation (time=2 ns) of all folds, in the case of K+ models (Figure S7 in File S3). 

To build C2, C3 and A1T3 mutated structures, we started from the mixed 2HY9 conformation, since the wt telomeric sequence showed a spectrum characteristic of a hybrid-type quadruplex; by contrast, as C1 sequence we used the NMR model 2KM3 reported by Lim et al. [23].

Each receptor was then placed in a cubic cell, with size adjusted to maintain a minimum distance of 25 Å to the cell boundary, and soaked with a pre-equilibrated box of water using the System Builder module of the Desmond package [40,41]. 

All overlapping solvent molecules were removed and a 0.05 M salt concentration of KCl was used in order to reproduce the experimental conditions. With the aim to evaluate the reliability of our protocol, we performed our simulations of the wt structures also in the presence of Na^+^, using it both as coordinating ion and in the 0.05 M NaCl buffer concentration. In order to optimize the geometries, all the receptors were energy minimized, using OPLS2005 [42,43] as force field. Starting from the energy minimized geometry, all the G-quadruplex folds were submitted to molecular dynamics simulations (MDs) under the following conditions: recording interval equal to 10 ps; 2 ns of simulation time at 350 K; pressure set to 1 bar; OPLS2005 as force field; a force restraint constant, used to fix the central coordinating ions, equal to 836.8 kJ/mol·Å and SPC water molecules. All simulations were performed by Desmond package. We intentionally chose a temperature of almost 15 K higher than the wt melting point to compensate the very small simulation time if compared to that of the experimental assay. 

Desmond simulation event analysis tool was used to monitor, over 2 ns, the Root Mean Square deviation (RMSd), calculated onto all the G-quadruplex atoms, and the frequency of the occurrence of the hydrogen bonds (HBs) in the guanine core, considering all the sampled MDs structures, for a total of 200 observations.

## Results

### Oligonucleotide design

The hTel oligonucleotide (5’-AGGGTTAGGGTTAGGGTTAGGG-3’), corresponding to the wild-type (wt) human telomeric sequence, contains four GGG repeats that form a G-quadruplex structure with three stacked G-quartets (Figure 1). The G-rich tracts are linked by TTA looping regions. The chosen hTel sequence had an additional A nucleotide at its 5’-end (wt, Table 1), according to an extensively studied model [7,9,27,44-46]. Mutations were introduced exclusively in the loop nucleotides; bases within the loops were named 1, 2 or 3, according to their 5’→3’ position. Bases at each position were substituted with a cytosine (C) in all three loops concurrently (oligonucleotides C1, C2, C3), or separately in each loop (oligonucleotides C1a, C1b, C1c, C2a, C2b, C2c, C3a, C3b, C3c, C3d). The TTA sequence within each loop was substituted with CCC, one loop at the time (oligonucleotides CCC1, CCC2, CCC3, Table 1). Adenines (As) at position 3 were also substituted with thymines (Ts) (oligonucleotide T3). Finally, As were moved to loop positions 1 or 2 (oligonucleotides A1T3 and A2T3, Table 1). 

**Table 1:** Mutant telomeric oligonucleotides used in this study.

**DNA name**	**5’-end**	**G-tract 1**	**Loop1**	**G-tract 2**	**Loop2**	**G-tract 3**	**Loop3**	**G-tract 4**
**wt**	A	GGG	TTA	GGG	TTA	GGG	TTA	GGG
**C1**	A	GGG	**C**TA	GGG	**C**TA	GGG	**C**TA	GGG
**C1a**	A	GGG	**C**TA	GGG	TTA	GGG	TTA	GGG
**C1b**	A	GGG	TTA	GGG	**C**TA	GGG	TTA	GGG
**C1c**	A	GGG	TTA	GGG	TTA	GGG	**C**TA	GGG
**C2**	A	GGG	T**C**A	GGG	T**C**A	GGG	T**C**A	GGG
**C2a**	A	GGG	T**C**A	GGG	TTA	GGG	TTA	GGG
**C2b**	A	GGG	TTA	GGG	T**C**A	GGG	TTA	GGG
**C2c**	A	GGG	TTA	GGG	TTA	GGG	T**C**A	GGG
**C3**	A	GGG	TT**C**	GGG	TT**C**	GGG	TT**C**	GGG
**C3a**	**C**	GGG	TTA	GGG	TTA	GGG	TTA	GGG
**C3b**	A	GGG	TT**C**	GGG	TTA	GGG	TTA	GGG
**C3c**	A	GGG	TTA	GGG	TT**C**	GGG	TTA	GGG
**C3d**	A	GGG	TTA	GGG	TTA	GGG	TT**C**	GGG
**CCC1**	A	GGG	**CCC**	GGG	TTA	GGG	TTA	GGG
**CCC2**	A	GGG	TTA	GGG	**CCC**	GGG	TTA	GGG
**CCC3**	A	GGG	TTA	GGG	TTA	GGG	**CCC**	GGG
**A1T3**	T	GGG	**A**T**T**	GGG	**A**T**T**	GGG	**A**T**T**	GGG
**A2T3**	T	GGG	T**AT**	GGG	T**AT**	GGG	T**AT**	GGG
**T3**	T	GGG	TT**T**	GGG	TT**T**	GGG	TT**T**	GGG

Mutated bases are in bold and underlined.

### Evaluation of the G-quadruplex conformations of the mutated telomeric sequences

The mutated oligonucleotides were initially assayed by circular dichroism spectroscopy (CD). This technique has been proved useful to discriminate a quadruplex topology from other generic folded structures [47]. Moreover, CD has been shown to identify the three different types of G-quadruplex topologies: in particular, parallel quadruplexes, belonging to group I, are identified by an intense positive CD peak at approximately 264 nm, a negative band at approximately 245 nm, and a negligible CD signal around 290 nm. Antiparallel quadruplexes (group III) show a positive band at 290 nm, a negative band at 264 and a positive peak at 240 nm; hybrid quadruplexes, which present both parallel and antiparallel strands (group II) display a positive band at 290 nm, a positive peak at 264 nm and a negative one at 240 nm [6,48]. Based on this spectroscopic behaviour, we characterized the topology of the loop mutated telomeric oligonucleotides. 

**Figure 2:**
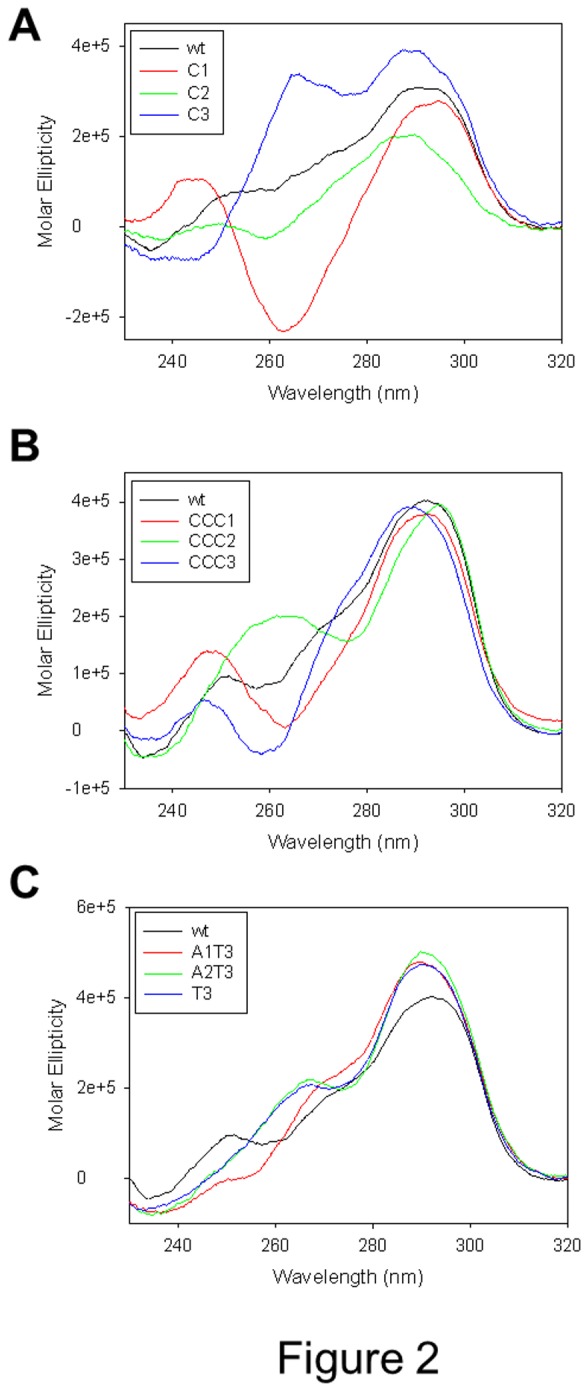
CD spectra of telomeric oligonucleotides mutated in the loop. A) Each oligonucleotide contained one single base mutated to C in each loop; B) each oligonucleotide contained the three nucleotides mutated to C in one loop; C) A bases in the loops were moved from the third to the second and first positions or mutated to T.

Each oligonucleotide (4 μM) was folded in the presence of 50 mM K^+^; CD spectra were measured and compared to that of the wild-type (wt) sequence. The wt telomeric sequence showed a spectrum with a maximum at 290 nm, a shoulder at 260 nm and a negative peak at 240 nm, which is characteristic of a hybrid-type quadruplex (black line, Figure 2A) [6,23]. The spectrum is likely the result of two hybrid conformations which have been reported to coexist in solution [12]. When mutating positions T1 in the loop with Cs (oligonucleotide C1), the resulting CD spectrum clearly depicted an antiparallel G-quadruplex (red line, Figure 2A). When Cs replaced positions T2, the spectroscopic signal displayed spectra less intense but very similar to that of the wt sequence (green line, Figure 2A). In contrast, oligonucleotide C3, where positions A3 were substituted by Cs, generated a CD spectrum with two intense positive bands at 290 and 264 nm and a negative peak at 245 nm that may indicate either a hybrid-type quadruplex configuration or the coexistence of different conformations. In terms of relative peak intensities, the acquired spectrum consistently differed from that obtained with the wt sequence (blue line, Figure 2A).

Replacement of the TTA loop sequence with CCC in the first (oligonucleotide CCC1) and in the third (oligonucleotide CCC3) loop shifted the original hybrid spectrum of the wt sequence to that of an antiparallel-like quadruplex (red and blue lines, respectively, Figure 2B); in contrast, substitution in the second loop (oligonucleotide CCC2) originated a spectrum characteristic of a hybrid-type structure (green line, Figure 2B).

Sequences where positions A3 were exchanged with positions T1 or T2 (oligonucleotides A1T3 and A2T3) maintained CD spectra very similar to that of the wt sequence (red and green lines, respectively, Figure 2C). Similarly, replacement of positions A3 with T (oligonucleotide T3) displayed again a hybrid-type CD signature (blue line, Figure 2C).

**Figure 3:**
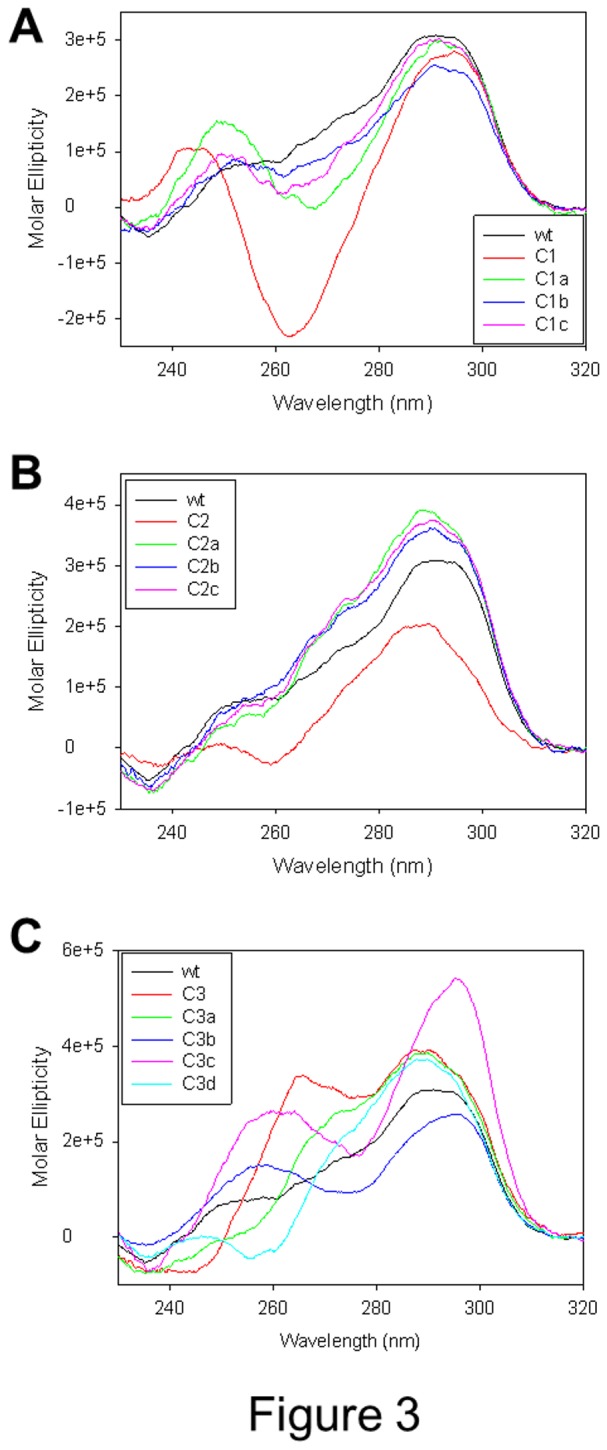
CD spectra of telomeric oligonucleotides mutated in the loop. In each oligonucleotide one single base was mutated.

To test the contribution of each mutation in oligonucleotides C1, C2 and C3, sequences where only one position in one loop was mutated at a time were assayed (oligonucleotides series C1, C2, C3, Table 1). As shown in Figure 3A, oligonucleotides C1a, C1b and C1c (lines, green, blue and magenta, respectively) displayed CD spectra with a major positive peak at 290 nm and shallow negative and positive peaks at 265 and 250 nm, respectively, very similar to the wt spectrum. In the case of the C2 series, all oligonucleotides mutated at a single base displayed spectra typical of a hybrid topology and very similar to that of the wt sequence (lines green, blue and magenta, Figure 3B). The C3 series exhibited the most heterogeneous behaviour: when the mutated base was at the very 5’-end of the oligonucleotide (C3a), the CD spectrum showed a maximum at 290 nm, a shoulder at 265 nm and a negative band at 240 nm, depicting a hybrid-type topology, similar to that of the wt sequence (Figure 3C, green line). When the mutation was at the 3’-end, in loop 3, the spectroscopic signal showed a positive peak at 290 nm, a shoulder at 265 nm, a negative peak at 260 nm and a positive band at 240 nm, indicating a mixture of antiparallel and hybrid topologies (Figure 3C, cyan line). Mutations in loops 2 (oligonucleotide C3b) and 3 (oligonucleotide C3c) resulted in spectra similar to that of the three-base-mutated sequence (C3), i.e. positive peaks at 290 and 260 nm, negative peak at 240 nm, but with different peak relative intensities (Figure 3C, blue and magenta lines).

**Figure 4:**
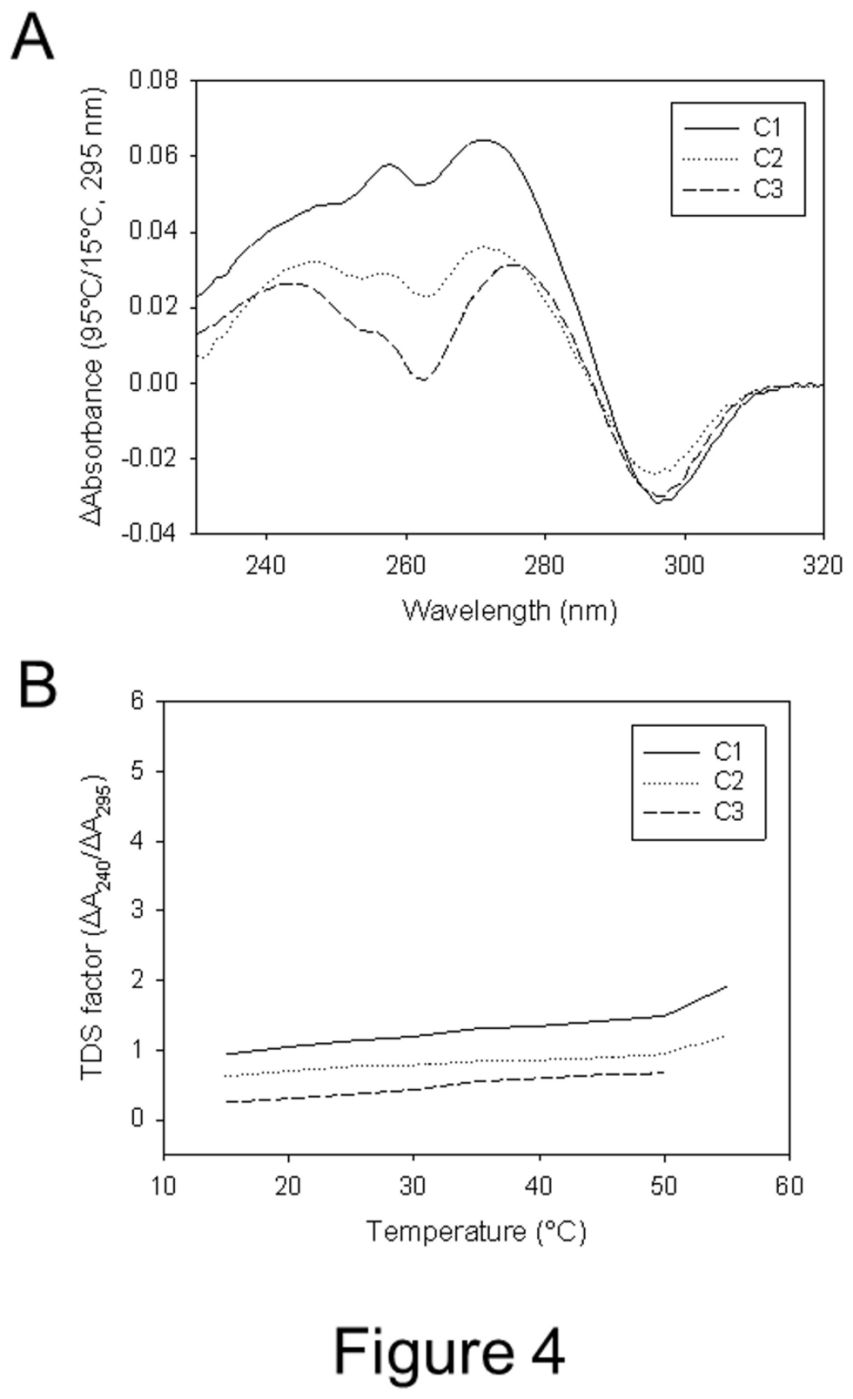
Thermal difference spectra (TDS) and TDS factor plots of three representative oligonucleotides. A) TDS of C1, C2 and C3 sequences; B) TDS factors of C1, C2 and C3 sequences. TDS and TDS factors of all oligonucleotides are available in Figure S8 in File S4.

UV absorption of the mutated oligonucleotides was next measured before and after melting to generate the thermal difference spectrum (TDS) which had been reported to provide a fingerprint of G-quadruplex groups [6]. All tested oligonucleotides displayed G-quadruplex characteristic TDS (Figure 4A and Figure S8A in File S4) and TDS factors (ΔA_240nm_/ΔA_295nm_) below 2 (Figure 4B and Figure S8B in File S4), indicative of group II and group III quadruplexes, therefore confirming correct assignment of CD signatures. 

**Figure 5:**
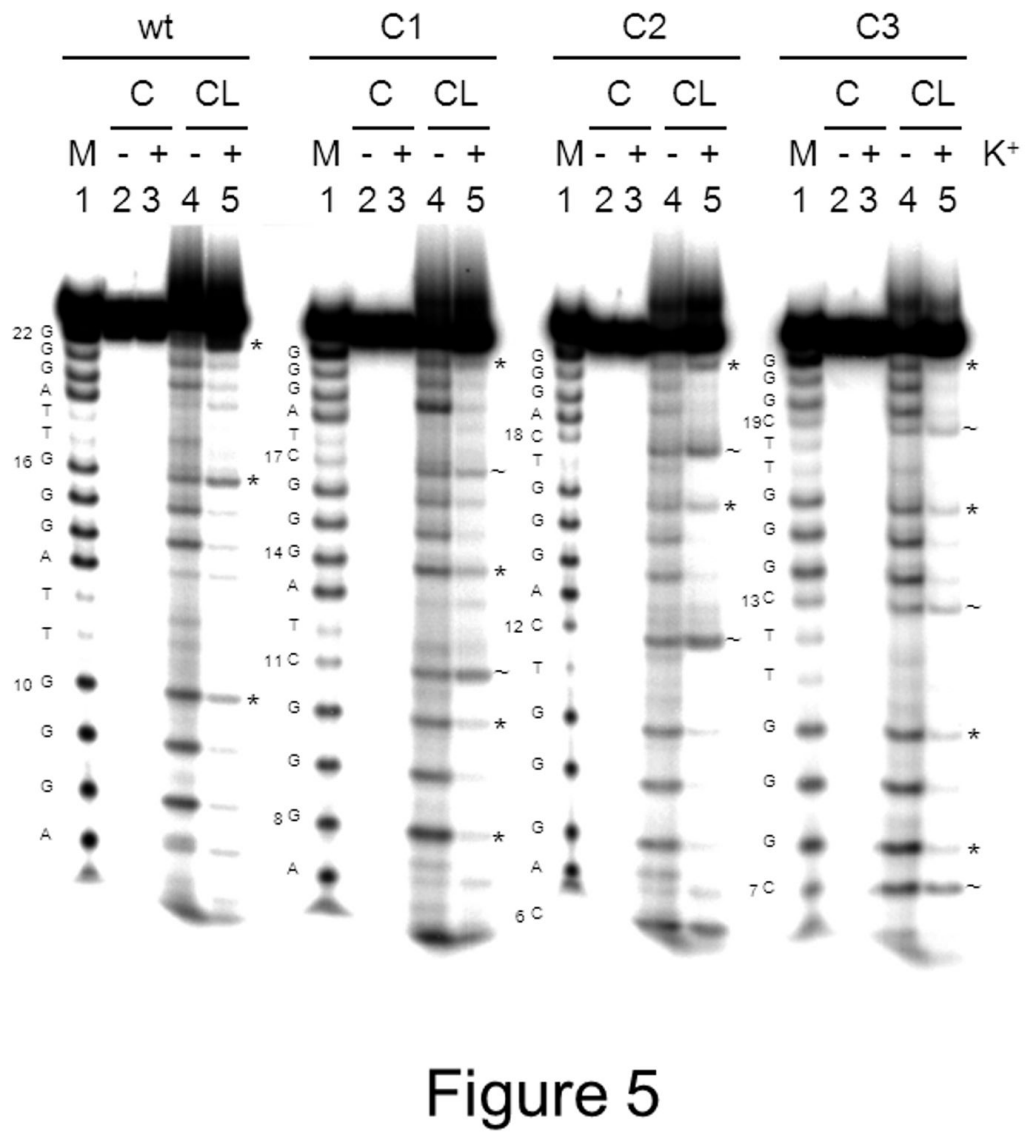
Clerocidin protection assay of the wt, C1, C2 and C3 telomeric oligonucleotides. Sequences were heat denatured, folded in the presence or absence of K^+^ and treated with CL followed by hot piperidine (CL lanes) or just treated with piperidine (C lanes). M indicates the marker lane obtained with the Maxam and Gilbert protocol. Base sequences are shown aside each gel image. Symbols * and ~ indicate protected G and C bases, respectively.

To acquire further details on the G-quadruplex folding of the wt, C1, C2 and C3 sequences, the folded oligonucleotides were tested on a clerocidin-based footprinting. Clerocidin is a natural molecule that alkylates single-stranded G (at N7), C (at N3) and A (at N1) bases and induces strand cleavage at G- and C-alkylated sites [49-53]. In principle, because N7 moieties of Gs in the G-quadruplex structure are involved in the Hoogsteen base pairing, necessary for G-quartet formation, they should not be available to clerocidin alkylation. However, Gs in the G-quadruplex may be differently tilted or buried according to the stretching or steric hindrance imparted by the linker regions, or may be exposed during the structural folding/refolding equilibria. While it is not possible to independently determine an unknown G-quadruplex conformation solely based on a footoprinting method, it is possible to indicate its similarity to a known conformation based on the conservation of the cleavage pattern. Based on these properties, we have shown that the human telomeric sequence in the presence of K^+^ (hybrid G-quadruplex structure) or Na^+^ (parallel G-quadruplex topology) are differently alkylated and therefore differentiated by clerocidin [53]. We have applied here the same method to confirm that the introduced loop mutations induced different conformations, as found by CD analysis. The two sequences that showed the highest degree of conformational diversity by CD with respect to the wt oligonucleotide, i.e. C1 and C3 were tested. C2 was additionally analyzed as representative of oligonucleotides with CD spectrum similar to that of the wt sequence. Clerocidin-mediated footprinting of the wt telomeric sequence showed exposure of G10, G16 and G22 (symbols *, lane 5 wt, Figure 5). These bases are involved in the two external G-quartets: stretching at these positions or folding/unfolding equilibria are likely the reason for the observed availability to clerocidin alkylation. In contrast, in C1, besides G10 and G22, other exposed bases with respect to the wt sequence were G8 and G14 (symbols *, lane 5 C1, Figure 5). This is in accordance with an antiparallel-like structure: in fact, the antiparallel conformation of the wt telomeric sequence obtained in Na^+^, showed the typical pattern of G10, G14 and G22 accessibility [53]. In addition, the introduced C bases present in the loops were also targeted by clerocidin, with C17 cleaved to a much lesser extent than C11 (symbols ~, lane 5 C1, Figure 5), which is in accordance with location of C11 in the loop and of C17 in the G:C:G:C tetrad [23]. The C2 sequence showed accessibility at G16 and G22, while G8-G10 were mainly protected. In contrast, all C bases were extremely exposed to the cleavage reaction, clearly indicating their presence in loop regions of the G-quadruplex structure (symbols * and ~ for exposed G and C bases, respectively, lane 5 C2, Figure 5). Finally, clerocidin-mediated cleavage pattern of C3 was different from any previous one: similar exposure was observed at G8, G10, G16 and G22 (see similar band intensity, symbols *, lane 5 C3, Figure 5). Interestingly, only C7 appeared largely accessible, while C13 and C19 were cleaved to an extent similar to that of G bases (compare bases indicated by symbols * and ~, lane 5 C3, Figure 5), pointing out to partial protection at these sites. Thus both G and C bases may concur to quartet formation, or C bases reside in linker regions that are buried within the tetraplex, therefore resulting partially protected from clerocidin. These data indicate that C3 adopts a hybrid-type folding pattern different from the other tested sequences.

### Stability of the mutated telomeric sequences by thermal analysis

**Figure 6:**
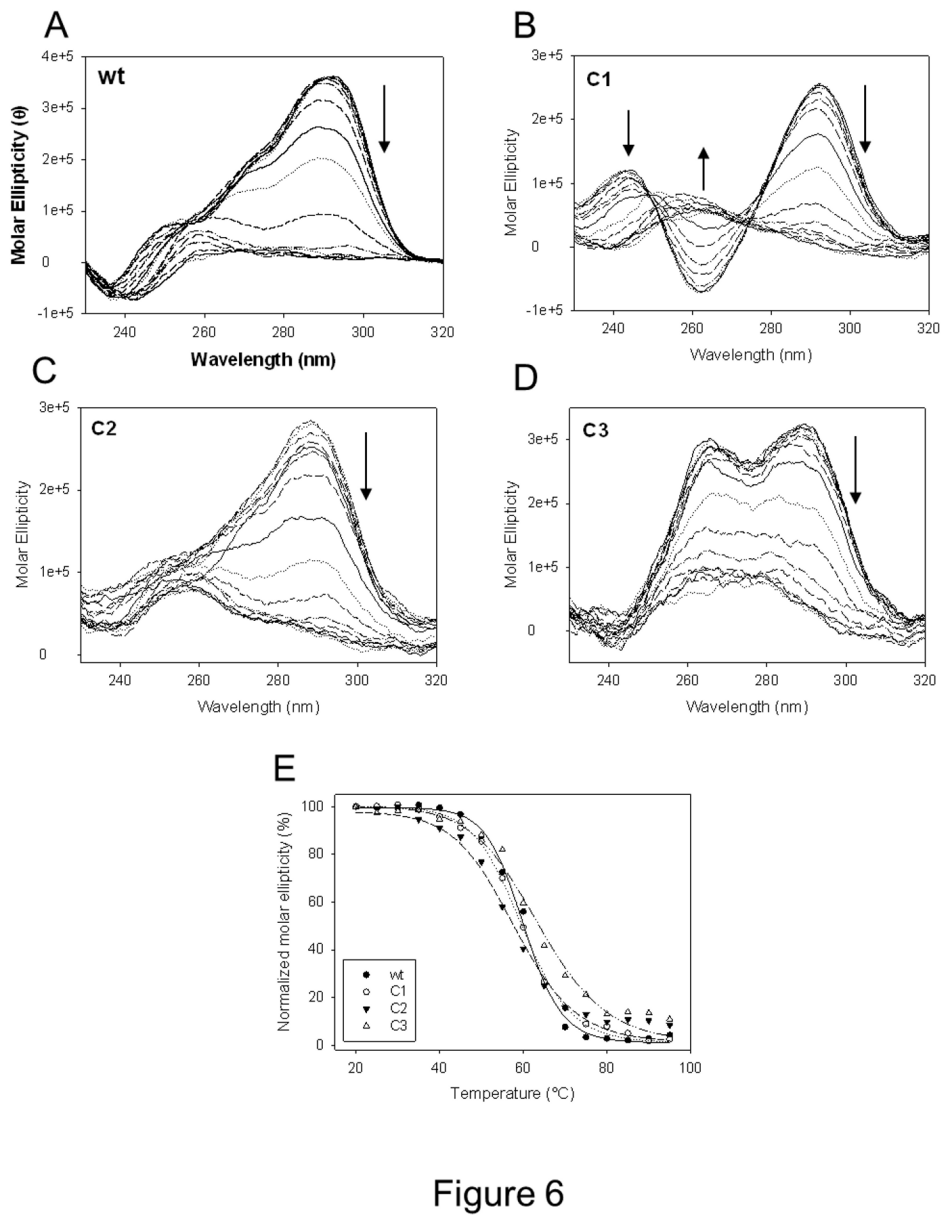
CD-monitored thermal denaturing assays of wt, C1, C2 and C3 telomeric oligonucleotides. A-D) CD spectra of each indicated oligonucleotide measured at increasing temperatures (20°C-95°). Arrows indicate curve trend at increasing temperatures. E) Molar ellipticities, measured at wavelength of maximum intensity, were plotted against temperature.

To determine the thermal stability of the mutated oligonucleotides, temperature of melting (T_m_) values were acquired by CD thermal unfolding experiments (Figure 6). In our conditions T_m_ of the wt sequence was 61.4°C (Table 2). For the C1 and C2 series, T_m_ values were similar but consistently lower than T_m_ of the wt sequence. Importantly, oligonucleotides with three mutated bases (C1 and C2) were less stable than their analogues with one single mutation (i.e. C1a, C1b, C1c, C2a, C2b, C2c). Strikingly in contrast, oligonucleotides of the C3 series were more stable than the wt sequence. In particular, oligonucleotides C3 and C3b, which displayed clear hybrid/mixed spectra, were stabilized to a higher extent (Table 2). Mutations of the three bases in each loop (in particular oligonucleotides CCC2 and CCC3), as well as exchange of TA positions (A1T3, A2T3) destabilized the G-quadruplex structure. The most prominent effect was observed for oligonucleotide A1T3, which was destabilized by 13.5°C (Table 2). 

**Table 2:** CD-acquired conformation and thermal stability of mutant telomeric oligonucleotides.

**sequence**	**topology at 20°C**	**T_m_ (290 nm**)	**ΔT_m_**
**wt**	**hybrid**	**61.4 ± 0.2**	-
**C1**	antiparallel	**59.5 ± 0.2**	-1.9
C1a	antiparallel/hybrid	60.7 ± 0.2	-0.7
C1b	antiparallel/hybrid	60.4 ± 0.2	-1.0
C1c	antiparallel/hybrid	61.0 ± 0.1	-0.4
**C2**	antiparallel/hybrid	**58.2 ± 0.2**	-3.2
C2a	hybrid = wt	59.4 ± 0.3	-2.0
C2b	hybrid = wt	59.9 ± 0.2	-1.5
C2c	hybrid = wt	60.1 ± 0.1	-1.3
**C3**	hybrid ≠ wt	**63.6 ± 0.2**	+2.2
C3a	hybrid = wt	62.2 ± 0.3	+0.8
C3b	hybrid ≠ wt	63.4 ± 0.1	+2.0
C3c	hybrid ≠ wt	62.5 ± 0.3	+1.1
C3d	antiparallel/hybrid	61.8 ± 0.2	+0.4
CCC1	antiparallel	60.8 ± 0.1	-0.6
CCC2	hybrid ≠ wt	59.0 ± 0.3	-2.4
CCC3	antiparallel	57.8 ± 0.2	-3.6
T3	hybrid = wt	60.5 ± 0.1	-0.9
A2T3	hybrid = wt	58.1 ± 0.1	-3.3
A1T3	hybrid = wt	47.9 ± 0.5	-13.5

T_m_ with standard deviation values are indicated.

### Stability of the mutated telomeric sequences by molecular dynamics simulations

In order to rationalize the most striking results obtained after the thermal stability analysis, we performed 2 ns molecular dynamics simulations (MDs) of the wt, C1, C2, C3 and A1T3 mutated conformations in the presence of the coordinating ion. First of all we aimed at reproducing the experimental conditions of the wt G-quadruplex sequence, including in our analysis 1KF1 [9], 143D [7] and 2HY9 [33] models as examples of parallel, antiparallel and mixed conformations. MDs were carried out using both K^+^ and Na^+^ as the coordinating ion for the three considered structures, finally evaluating the Root Mean Square deviation, calculated on all atoms. Specifically, as reported in the literature [7-9], we obtained a lower RMSd average value in the presence of K^+^ if compared to Na^+^ for the parallel and mixed folds; by contrast, we found a higher stabilization in the presence of Na^+^ for the antiparallel conformation. Thus, since our computational protocol was able to well reproduce the experimental observations, we applied it to the C1 mutated sequence, in order to further validate our approach. In particular, we started from the NMR model 2KM3 reported by Lim et al. [23] and we checked the stabilizing effect mediated by K^+^ and Na^+^ ion onto the G-quadruplex chair-type conformation. In the presence of K^+^ we obtained an average RMSd equal to 4.28 Å, while in the presence of Na^+^ we observed a reduced stabilization (RMSd equal to 4.85 Å), which is in accordance with the experimental data. 

The molecular dynamics experiments of C2, C3 and A1T3 mutated sequences were performed using only K^+^ as the coordinating ion, considering that each oligonucleotide was folded in the presence of 50 mM K^+^. As shown in Table 3, in agreement with the melting data, we found that C3 structure was associated with the lowest average RMSd value, indicating its highest conformational stabilization among all the studied G-quadruplex structures. By contrast, when analysing the A1T3 mutated sequence, we observed the maximum calculated RMSd, confirming its strong destabilizing effect with respect to the wt sequence (ΔT_m_ equal to -13.5°C). MDs of C1 and C2 sequences indicated similar RMSd average values, but always related to a reduced stabilization if compared to that of the wt structure. Such an observation is consistent with C1 and C2 temperatures of melting, that resulted lower than T_m_ of the wt conformation.

**Table 3:** RMSd values, calculated on all atoms, of wt and mutated telomeric oligonucleotides obtained after 2 ns molecular dynamics simulations.

**Model**	**RMSd**	
	**K^+^**	**Na^+^**
**1KF1 (parallel)**	3.20	3.70
**143D (antiparallel)**	2.82	2.70
**2HY9 (mixed)**	3.19	4.32
**C1**	4.28	4.85
**C2**	4.65	
**C3**	2.40	
**A1T3**	4.90	

The reported values are expressed in Å.

**Figure 7:**
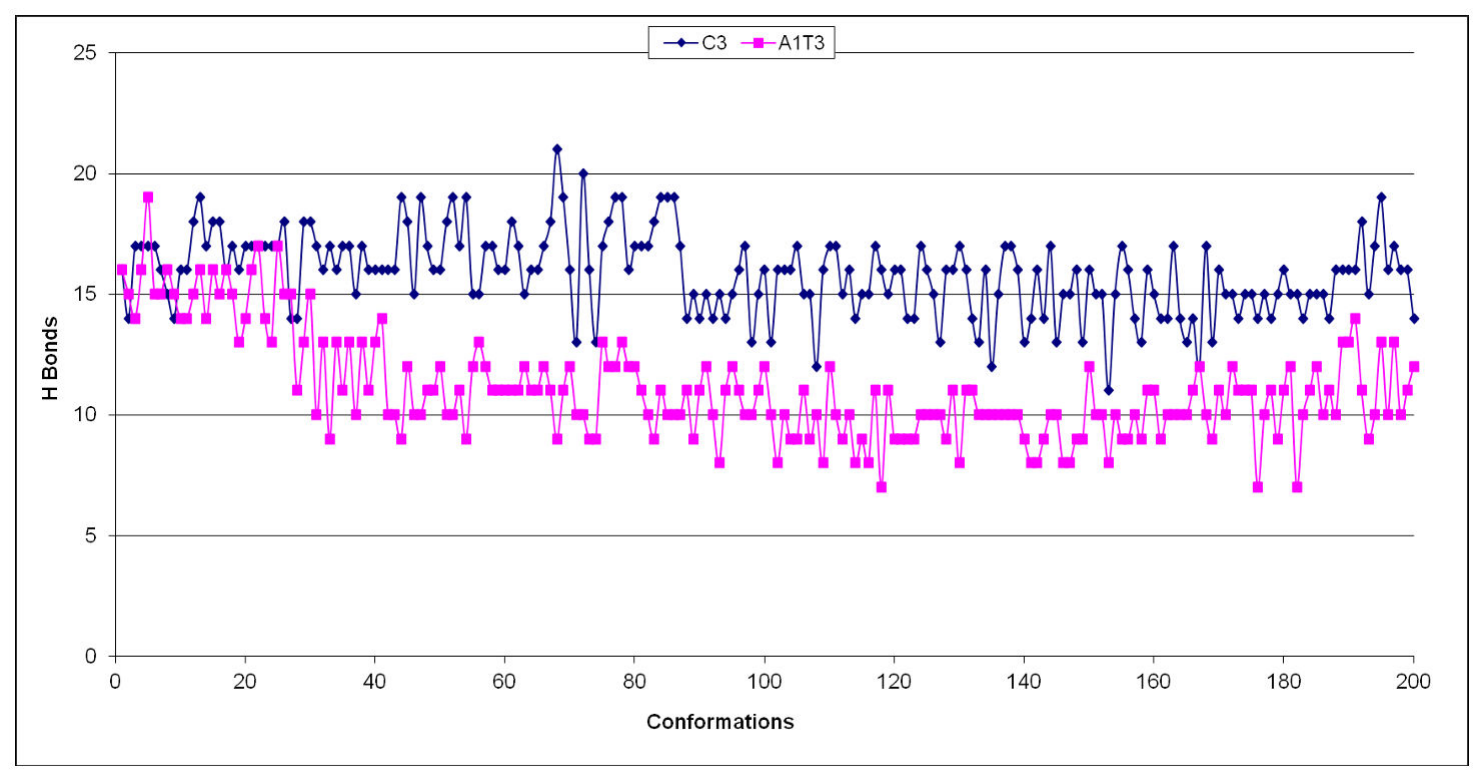
Frequency of the occurrence of the hydrogen bonds. Frequency of the occurrence of the hydrogen bonds (HBs), monitored over 2 ns, every 10 ps, in the guanine core in the presence of C3 and A1T3 mutated G-quadruplex sequences.

**Figure 8:**
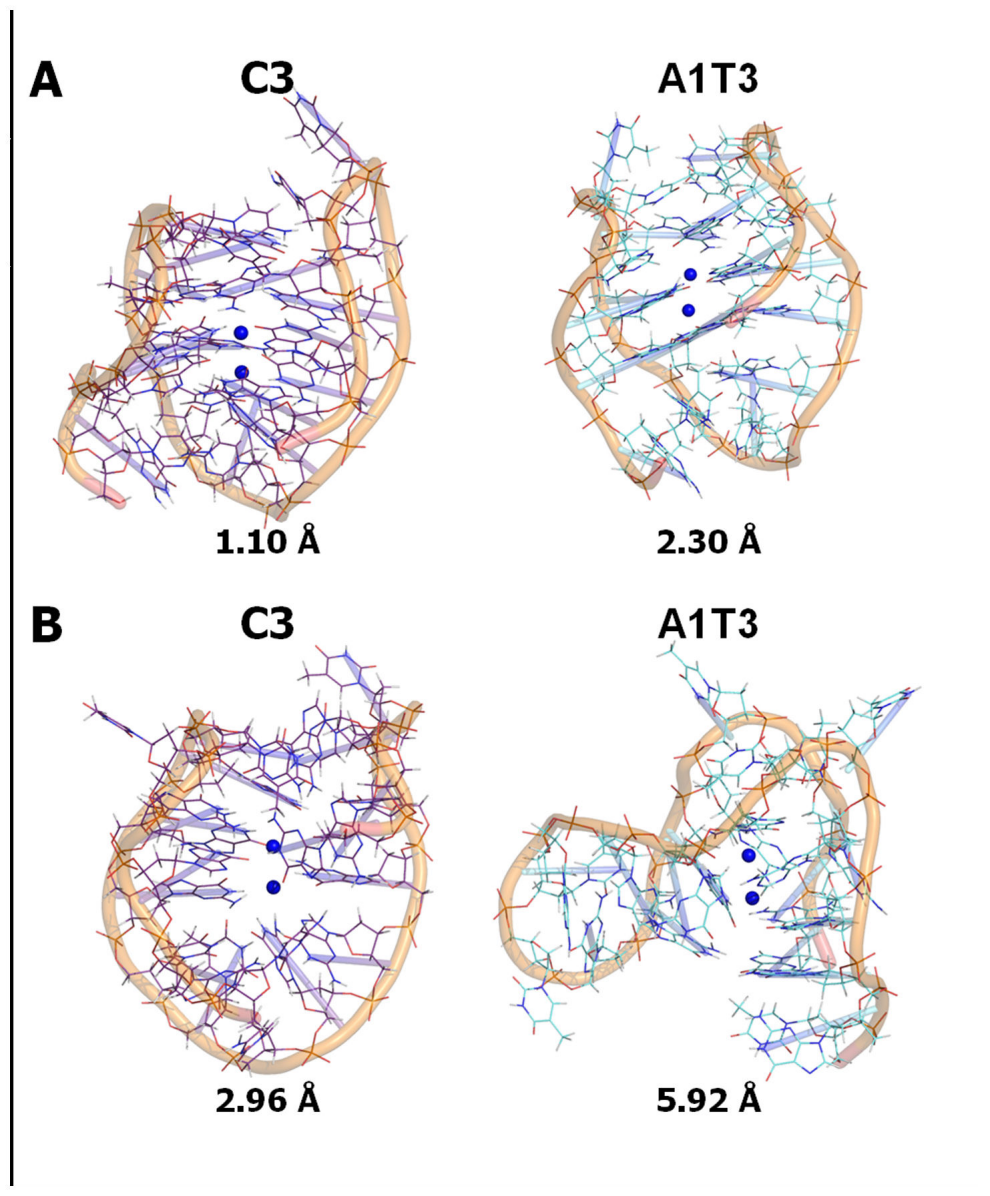
RMSd conformation of C3 and A1T3 mutated G-quadruplex sequences. Lowest (A) and highest (B) RMSd conformation of C3 and A1T3 mutated G-quadruplex sequences during 2 ns MD simulations with respect to the starting structure (2HY9). The DNA is shown in purple (C3) and aquamarine (A1T3) wireframe rendering and its strand as orange cartoon. The K^+^ coordinating ions are represented as blue spheres. The RMSd value of each conformation is reported and expressed in Å.

With the aim to better rationalize the MDs results of the most and less stable C3 and A1T3 mutated sequences, respectively, we monitored over 2 ns, every 10 ps (for 200 total observations), the frequency of the occurrence of the hydrogen bonds (HBs) in the guanine core. Interestingly, as reported in Figure 7, the number of occurrences of HBs in the presence of C3 was significantly higher than that in the presence of A1T3 conformation (3174 *versus* 2225 occurrences). Such a finding further confirmed C3 theoretical best stabilization with respect to the analyzed G-quadruplex sequences, according to the experimental observations. The structures of C3 and A1T3 are reported in Figure 8 and indicate the lowest (panel A) and the highest (panel B) RMSd conformations during 2 ns MD simulations with respect to the starting structure (2HY9). As highlighted by the average RMSd analysis (Table 3), in C3 conformation, integrity of the guanine core was more preserved if compared to that of A1T3. In particular for the C3 sequence, the lowest calculated RMSd value was equal to 1.10 Å, while for the A1T3 mutant was 2.30 Å (Figure 8A); analysing these structures, obtained from the initial frames of the simulation, the global number of HBs in the guanine core was almost similar (16 in C3 *vs.* 12 in A1T3). By contrast, as shown in Figure 8B, the C3 conformation associated to the highest RMSd value (2.96 Å) was more conserved with respect to that of A1T3 mutated sequence (5.92 Å), as confirmed by its higher number of HBs in the guanine core if compared to that of A1T3 (12 in C3 *vs.* 6 in A1T3). 

Since computational models reported in the literature present a third coordinating cation, located between the terminal quartet and the A:A diad [54-56], we also performed MDs using three K^+^/Na^+^ ions (data not shown). Interestingly, the same stabilization trend was observed if compared to that in presence of two cations.

## Discussion

By performing systematic mutations in the loops of the human telomeric sequence, we analyzed the effect of each base variation on the telomeric G-quadruplex prevalent topologies and associated stabilities.

When positions T1 in the three loops were substituted with Cs, shifting from the hybrid structure of the wt sequence to a prevalent antiparallel conformation was found. The C1 oligonucleotide had already been described [23,24,27]. In particular, an attentive NMR analysis revealed an unexpected G-quadruplex formed by two G•G•G•G tetrads and one G•C•G•C tetrad with an overall antiparallel topology. This particular structure was confirmed by the current CD and footprinting data: the presence of C at positions 1 in the first and third loop conserved the strong antiparallel conformation and Cs involved in the G•C•G•C tetrad formation were protected from clerocidin alkylation; in contrast, introduction of C at position 1 in just one of the three loops, prevented formation of the antiparallel structure. However, especially in the case of oligonucleotide C1a, the resulting conformation still resembled that of an antiparallel quadruplex but with less intense bands: therefore, it is likely that more than one conformation coexists in solution and that an alternative G•C•G•G tetrad forms, incrementing the antiparallel population. When positions T2 were substituted with Cs, minor changes in the CD spectra were observed. When all three loops were involved in the mutation, a shift to a shallow antiparallel-like spectrum may indicate the presence of multiple antiparallel-hybrid conformations. In both T1 and T2 substituted oligonucleotides, the G-quadruplex structures were slightly but consistently less stable than the wt sequence. In particular, oligonucleotides containing three mutations (C1 and C2) were the most severely destabilized. In striking contrast, the C3 sequence provided a CD spectrum that, while being typical of a hybrid topology, largely differed from that obtained with the wt sequence in terms of relative peak intensity. In particular, the band at 260 nm was very pronounced and similar in intensity to that at 290 nm and a shallow negative band at 275 nm could be observed. This type of spectrum had already been reported for G-quadruplex topologies, such as the d(G_3_TG_3_T_4_G_3_T_3_G_3_) sequence in [6]. Interestingly, mutation of just one base in the first or second loop still produced dramatic effects in terms of spectroscopic signal which remained similar to that of oligonucleotide C3. Even more intriguingly, the C3 series of oligonucleotides was moderately but constantly more stable than the wt sequence: the oligonucleotides that reported two distinct bands at 260 and 290 nm and a shallow negative band at 270 nm (C3, C3b and C3c) furnished higher T_m_ values. The clerocidin-mediated footprinting analysis confirmed a C3 conformation different from that of the wt sequence: in particular, two out of three Cs were partially protected from cleavage, indicating that either both G and C bases concur to quartet formation, or C bases reside in linker regions that are buried within the tetraplex. 

When the TTA sequence in the first and third loop, separately, was substituted with CCC, the oligonucleotides folded in an antiparallel conformation which was probably the result of the presence of C in position 3 of the loops which allowed the formation of the G•C•G•G and G•G•G•C tetrads as in oligonucleotides C1a and C1c [23]. Oppositely, when CCC was placed in the second loop, the oligonucleotide adopted a hybrid conformation, indicating that mutation in the second loop induced a less intense perturbation of the original wt topology. Finally, exchange of position A3 with positions T1 or T2 (A1T3, A2T3), and substitution of position A3 with T (T3) generated oligonucleotides that maintained a hybrid structure, therefore displaying little conformational variation with respect to the wt sequence topology. However, it is interesting to note that stability of the resulting G-quadruplex structures was significantly affected, especially in the case of A1T3. This confirms that A in the first position of the loop is detrimental to quadruplex stability, as reported in [25].

Our computational approach was able to well reproduce the experimental data, allowing us to rationalize the conformational perturbations observed in the mutated sequences: an increased or decreased possibility to establish hydrogen bonds is responsible for the observed varied stabilities of oligonucleotides C3 and A1T3, respectively.

Based on these results, the following conclusions can be extrapolated: 1) Substitution of positions A3 with Cs increases stability of the G-quadruplex which adopts an original hybrid topology. In contrast, substitution of positions A3 with Ts does not significantly affect G-quadruplex topology and stability. Therefore, both low steric hindrance (such as that of pyrimidines, C and T) and hydrogen donor groups (i.e. NH_2_ in C and A) are features that may concur and have comparable weight on G-quadruplex stabilization. In C3 a higher degree of stabilization was observed because both factors were present. 2) Substitution of positions T1 with Cs significantly perturbs the wt hybrid conformation and shifts the stability towards an antiparallel form which is moderately less stable. In this case the presence of an unconventional tetrad is probably the cause of the observed lower stability (reported also in [23]). Substitution with the bulkier A profoundly destabilizes the G-quadruplex (confirming previous observations [25]). 3) Substitution of positions T2 with Cs does not significantly perturb the original hybrid conformation, but consistently decreases G-quadruplex stability. Thus, a hydrogen acceptor rather than a hydrogen bond donor moiety (present at the 4 position of T and C respectively) is preferred in this loop location. In terms of quadruplex stability, a role could also be played by the hydrophobic methyl substituent present at position 5 of T. Therefore, the overall stability of the wt G-quadruplex is favoured by proton acceptor groups at position T1 and T2, while at position A3 proton donor groups must be present. A lower steric hindrance at position 3 of the loop would augment overall quadruplex stability. We have also shown that the C3 sequence adopts a G-quadruplex conformation which is different from the wt hybrid form and from the C1 antiparallel configuration. Based on the CD spectra, fully parallel and antiparallel configuration can be excluded, and thus a hybrid topology different from the reported forms 1 and 2 is plausible. Multiple topologies due to folding equilibria operating on a single sequence may also be present, with CD spectra depicting the most prevalent one. 

The present work reveals important biological implications: mutations that we have found to affect G-quadruplex topology and stability were reported to display the most relevant pathologic effect; in fact, the C1 mutation has been associated with high mutation rates in the male germline, while C2 mutation did not show the same level of instability [4]. Therefore, the DNA G-quadruplex conformational knowledge at the telomere level has important therapeutic implications: i) analysis of the mutated sequence stability and topology may anticipate the severity of the pathologic effect; ii) conformational deconvolution of the mutated sequence by computational methods may help the rational design of drugs acting at the telomeric level. To this end, the developed computational protocol is a very useful tool for the prediction of new G-quadruplex oligonucleotides stability and, consequently, for the rational design of their selective binders.

In conclusion we have shown structural modifications of the telomeric sequence as a function of loop composition. This information has to be taken into account when rationally designing telomeric G-quadruplex ligands.

## Supporting Information

File S1 **Table S1, Internal energies, expressed in KJ/mol, of the experimental and energy minimized structures of G4 2HY9 model.** **Table** **S2**, Internal energies, expressed in KJ/mol, of the experimental and energy minimized structures of G4 2JPZ model. **Table** **S3**, Internal energies, expressed in KJ/mol, of the experimental and energy minimized structures of G4 2JSL model. **Table** **S4**, Internal energies, expressed in KJ/mol, of the experimental and energy minimized structures of G4 2JSM model.
(DOCX)

File S2 **Figure S1, Monitoring graph of 8 dummy angles between the guanine core and K^+^ coordinating cations after 2ns of molecular dynamics of 1KF1 structure.** On the x- and on the y-axes are reported, respectively, the sampled observations and the angle values expressed in deg. K1, K2, K3, K4, K5, K6, K7 and K8 are defined, respectively, with the following atoms: N7-G4/K1/N7-G16, N7-G10/K1/N7-G22, N7-G9/K1/N7-G21, N7-G3/K1/N7-G15, N7-G3/K2/N7-G15, N7-G9/K2/N7-G21, N7-G2/K2/N7-G14, N7-G8/K2/N7-G20. **Figure S2**, Monitoring graph of 8 dummy angles between the guanine core and Na^+^ coordinating cations after 2ns of molecular dynamics of 1KF1 structure. On the x- and on the y-axes are reported, respectively, the sampled observations and the angle values expressed in deg. Na1, Na2, Na3, Na4, Na5, Na6, Na7 and Na8 are defined, respectively, with the following atoms: N7-G4/Na1/N7-G16, N7-G10/Na1/N7-G22, N7-G9/Na1/N7-G21, N7-G3/Na1/N7-G15, N7-G3/Na2/N7-G15, N7-G9/Na2/N7-G21, N7-G2/Na2/N7-G14, N7-G8/Na2/N7-G20. **Figure S3**, Monitoring graph of 8 dummy angles between the guanine core and K^+^ coordinating cations after 2ns of molecular dynamics of 143D structure. On the x- and on the y-axes are reported, respectively, the sampled observations and the angle values expressed in deg. K1, K2, K3, K4, K5, K6, K7 and K8 are defined, respectively, with the following atoms: N7-G4/K1/N7-G20, N7-G8/K1/N7-G16, N7-G3/K1/N7-G21, N7-G9/K1/N7-G15, N7-G3/K2/N7-G21, N7-G9/K2/N7-G15, N7-G2/K2/N7-G22, N7-G3/K2/N7-G21. **Figure S4**, Monitoring graph of 8 dummy angles between the guanine core and Na^+^ coordinating cations after 2ns of molecular dynamics of 143D structure. On the x- and on the y-axes are reported, respectively, the sampled observations and the angle values expressed in deg. Na1, Na2, Na3, Na4, Na5, Na6, Na7 and Na8 are defined, respectively, with the following atoms: N7-G4/Na1/N7-G20, N7-G8/Na1/N7-G16, N7-G3/Na1/N7-G21, N7-G9/Na1/N7-G15, N7-G3/Na2/N7-G21, N7-G9/Na2/N7-G15, N7-G2/Na2/N7-G22, N7-G3/Na2/N7-G21. **Figure S5**, Monitoring graph of 8 dummy angles between the guanine core and K^+^ coordinating cations after 2ns of molecular dynamics of 2HY9 structure. On the x- and on the y-axes are reported, respectively, the sampled observations and the angle values expressed in deg. K1, K2, K3, K4, K5, K6, K7 and K8 are defined, respectively, with the following atoms: N7-G6/K1/N7-G16, N7-G12/K1/N7-G24, N7-G5/K1/N7-G17, N7-G11/K1/N7-G23, N7-G5/K2/N7-G17, N7-G11/K2/N7-G23, N7-G4/K2/N7-G18, N7-G10/K2/N7-G22. **Figure S6**, Monitoring graph of 8 dummy angles between the guanine core and Na^+^ coordinating cations after 2ns of molecular dynamics of 2HY9 structure. On the x- and on the y-axes are reported, respectively, the sampled observations and the angle values expressed in deg. Na1, Na2, Na3, Na4, Na5, Na6, Na7 and Na8 are defined, respectively, with the following atoms: N7-G6/Na1/N7-G16, N7-G12/Na1/N7-G24, N7-G5/Na1/N7-G17, N7-G11/Na1/N7-G23, N7-G5/Na2/N7-G17, N7-G11/Na2/N7-G23, N7-G4/Na2/N7-G18, N7-G10/Na2/N7-G22.
(DOCX)

File S3 **Figure S7, Representation of DNA G4 stem, generated from the last snapshot of the molecular dynamics simulation (time=2 ns) of (**A**) parallel, (**B**) anti-parallel and (**C**) hybrid-1 conformations in the case of K^+^ models.** The DNA structure is colored by atom types with the coordinating K^+^ ions shown as magenta van der Waals spheres.
(DOCX)

File S4 **Figure S8, (A) Thermal difference spectra (TDS) and (B) TDS factor plots of all telomere mutant oligonucleotides studied in this work.**
(DOCX)
