# Supplementary material for: Conformation and Stability of Intramolecular Telomeric G-Quadruplexes: Sequence Effects in the Loops
Source: PLoS One. 2013 Dec 18;8(12):e84113. doi: 10.1371/journal.pone.0084113 (PMC3867476; doi:10.1371/journal.pone.0084113)
Supplement: File S1 — Table S1, Internal energies, expressed in KJ/mol, of the experimental and energy minimized structures of G4 2HY9 model. Table S2, Internal energies, expressed in KJ/mol, of the experimental and energy minimized structures of G4 2JPZ model. Table S3, Internal energies, expressed in KJ/mol, of the experimental and energy minimized structures of G4 2JSL model. Table S4, Internal energies, expressed in KJ/mol, of the experimental and energy minimized structures of G4 2JSM model. (DOCX) [file pone.0084113.s001.docx]

**Table S1.**

| **2HY9** | | |
| --- | --- | --- |
| # Structures | Experimental | Energy Minimized |
| 1 | -5423.40 | -15731.20 |
| 2 | -2519.49 | -15870.65 |
| 3 | -3714.97 | -15785.05 |
| 4 | -3816.30 | -15757.16 |
| 5 | -4170.11 | -15769.25 |
| 6 | -1739.08 | -15889.09 |
| 7 | -7185.60 | -15765.98 |
| 8 | -1382.14 | -15819.19 |
| 9 | -3418.09 | -15812.57 |
| 10 | -7671.31 | -15840.25 |

**Table S2.**

| **2JPZ** | | |
| --- | --- | --- |
| # Structures | Experimental | Energy Minimized |
| 1 | -6405.53 | -15795.89 |
| 2 | -2029.57 | -15785.14 |
| 3 | -8631.80 | -15850.78 |
| 4 | -6252.56 | -15852.60 |
| 5 | -8682.63 | -15783.66 |
| 6 | -9501.91 | -15858.96 |
| 7 | -8083.14 | -15858.79 |
| 8 | -7748.13 | -15859.97 |
| 9 | -9888.67 | -15780.24 |
| 10 | -8897.62 | -15767.40 |

**Table S3.**

| **2JSL** | | |
| --- | --- | --- |
| # Structures | Experimental | Energy Minimized |
| 1 | -13894.63 | -15756.44 |
| 2 | -13990.79 | -15774.88 |
| 3 | -13453.49 | -15793.35 |
| 4 | -13431.28 | -15732.97 |
| 5 | -13792.51 | -15795.62 |
| 6 | -13942.23 | -15838.37 |
| 7 | -13763.33 | -15823.16 |
| 8 | -14070.86 | -15732.01 |
| 9 | -13778.94 | -15787.61 |
| 10 | -13790.48 | -15842.68 |

**Table S4.**

| **2JSM** | | |
| --- | --- | --- |
| # Structures | Experimental | Energy Minimized |
| 1 | -14019.63 | -15882.28 |
| 2 | -14066.17 | -15765.97 |
| 3 | -13954.50 | -15773.30 |
| 4 | -14000.41 | -15842.40 |
| 5 | -14009.25 | -15776.06 |
| 6 | -14021.34 | -15842.90 |
| 7 | -13990.99 | -15763.57 |
| 8 | -14003.32 | -15872.14 |
| 9 | -14007.89 | -15873.66 |
| 10 | -14003.77 | -15786.67 |
