# Supplementary material for: Conformation and Stability of Intramolecular Telomeric G-Quadruplexes: Sequence Effects in the Loops
Source: PLoS One. 2013 Dec 18;8(12):e84113. doi: 10.1371/journal.pone.0084113 (PMC3867476; doi:10.1371/journal.pone.0084113)
Supplement: File S2 — Figure S1, Monitoring graph of 8 dummy angles between the guanine core and K+ coordinating cations after 2ns of molecular dynamics of 1KF1 structure. On the x- and on the y-axes are reported, respectively, the sampled observations and the angle values expressed in deg. K1, K2, K3, K4, K5, K6, K7 and K8 are defined, respectively, with the following atoms: N7-G4/K1/N7-G16, N7-G10/K1/N7-G22, N7-G9/K1/N7-G21, N7-G3/K1/N7-G15, N7-G3/K2/N7-G15, N7-G9/K2/N7-G21, N7-G2/K2/N7-G14, N7-G8/K2/N7-G20. Figure S2, Monitoring graph of 8 dummy angles between the guanine core and Na+ coordinating cations after 2ns of molecular dynamics of 1KF1 structure. On the x- and on the y-axes are reported, respectively, the sampled observations and the angle values expressed in deg. Na1, Na2, Na3, Na4, Na5, Na6, Na7 and Na8 are defined, respectively, with the following atoms: N7-G4/Na1/N7-G16, N7-G10/Na1/N7-G22, N7-G9/Na1/N7-G21, N7-G3/Na1/N7-G15, N7-G3/Na2/N7-G15, N7-G9/Na2/N7-G21, N7-G2/Na2/N7-G14, N7-G8/Na2/N7-G20. Figure S3, Monitoring graph of 8 dummy angles between the guanine core and K+ coordinating cations after 2ns of molecular dynamics of 143D structure. On the x- and on the y-axes are reported, respectively, the sampled observations and the angle values expressed in deg. K1, K2, K3, K4, K5, K6, K7 and K8 are defined, respectively, with the following atoms: N7-G4/K1/N7-G20, N7-G8/K1/N7-G16, N7-G3/K1/N7-G21, N7-G9/K1/N7-G15, N7-G3/K2/N7-G21, N7-G9/K2/N7-G15, N7-G2/K2/N7-G22, N7-G3/K2/N7-G21. Figure S4, Monitoring graph of 8 dummy angles between the guanine core and Na+ coordinating cations after 2ns of molecular dynamics of 143D structure. On the x- and on the y-axes are reported, respectively, the sampled observations and the angle values expressed in deg. Na1, Na2, Na3, Na4, Na5, Na6, Na7 and Na8 are defined, respectively, with the following atoms: N7-G4/Na1/N7-G20, N7-G8/Na1/N7-G16, N7-G3/Na1/N7-G21, N7-G9/Na1/N7-G15, N7-G3/Na2/N7-G21, N7-G9/Na2/N7-G15, N7-G2/Na2/N7-G22, N7-G3/Na2/ [file pone.0084113.s002.docx]

**Figure S1.**

**Figure S2.**

**Figure S3.**

**Figure S4.**

**Figure S5.**

**Figure S6.**
