# Supplementary figures and images for: Conformation and Stability of Intramolecular Telomeric G-Quadruplexes: Sequence Effects in the Loops
Source: PLoS One. 2013 Dec 18;8(12):e84113. doi: 10.1371/journal.pone.0084113 (PMC3867476; doi:10.1371/journal.pone.0084113)

**Figure S7.**


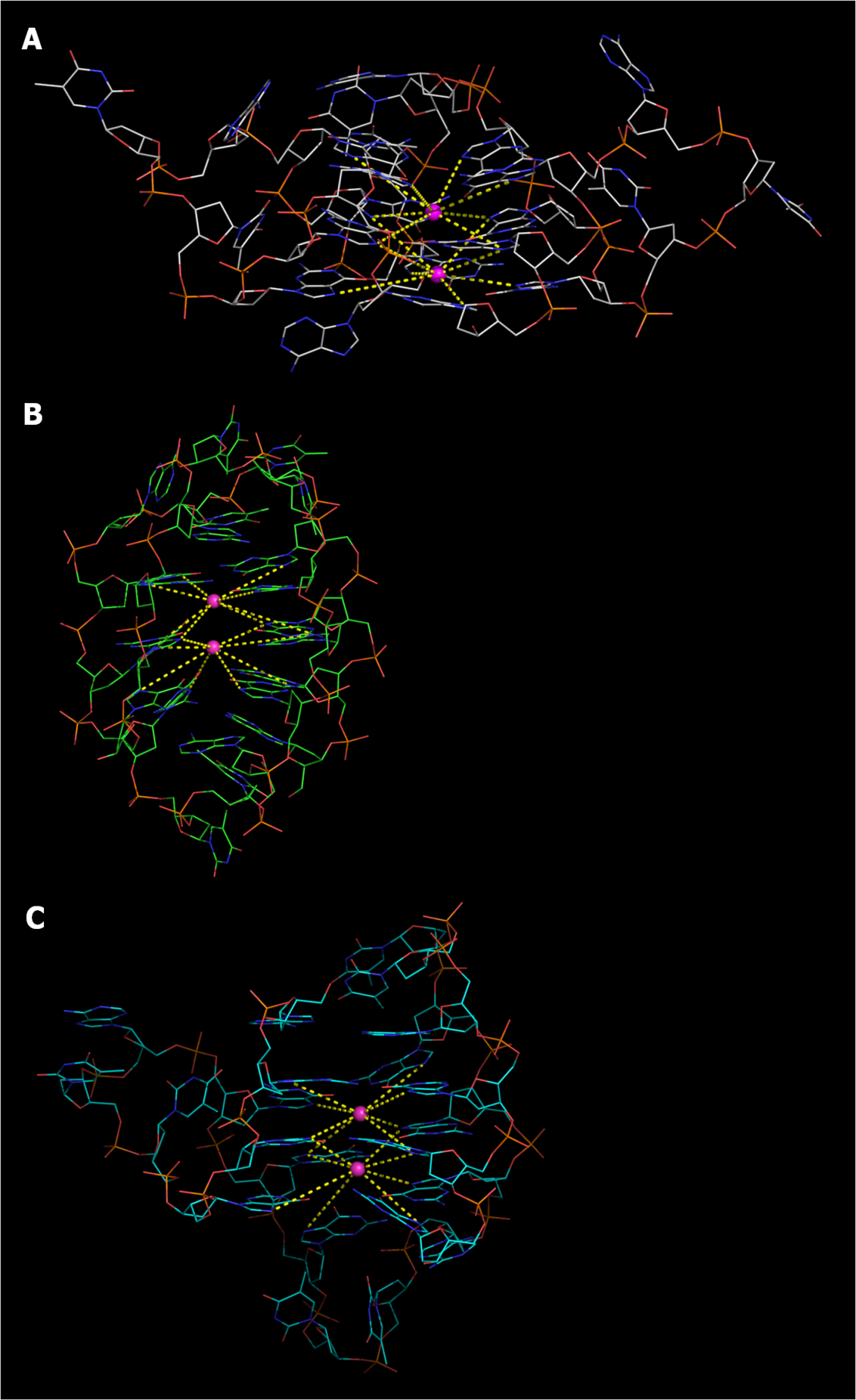

Supplement: File S3 — Figure S7, Representation of DNA G4 stem, generated from the last snapshot of the molecular dynamics simulation (time=2 ns) of (A) parallel, (B) anti-parallel and (C) hybrid-1 conformations in the case of K+ models. The DNA structure is colored by atom types with the coordinating K+ ions shown as magenta van der Waals spheres. (DOCX) [file pone.0084113.s003.docx]
