# Supplementary material for: Conformation and Stability of Intramolecular Telomeric G-Quadruplexes: Sequence Effects in the Loops
Source: PLoS One. 2013 Dec 18;8(12):e84113. doi: 10.1371/journal.pone.0084113 (PMC3867476; doi:10.1371/journal.pone.0084113)
Supplement: File S4 — Figure S8, (A) Thermal difference spectra (TDS) and (B) TDS factor plots of all telomere mutant oligonucleotides studied in this work. (DOCX) [file pone.0084113.s004.docx]

**Figure S8.**

**A**

**B**
